# Artificial Intelligence for Automated Continuous Quantification of Left Ventricular Ejection Fraction From Standard Adult Transthoracic Echocardiography: A Systematic Review

**DOI:** 10.7759/cureus.118446

**Published:** 2026-10-23

**Authors:** Aniket Dembi, Hilary Patankar, Simran Dhadda, Amer Hasanovic

**Affiliations:** 1 Haematology, University Hospitals of Derby and Burton NHS Foundation Trust, Derby, GBR; 2 Emergency Medicine, East and North Hertfordshire Teaching NHS Trust, Stevenage, GBR; 3 Oncology, University Hospitals of Derby and Burton NHS Foundation Trust, Derby, GBR; 4 Obstetrics and Gynaecology, Chesterfield Royal Hospital NHS Foundation Trust, Chesterfield, GBR

**Keywords:** artificial intelligence (ai), cardiac imaging techniques, left ventricular ejection fraction, lvef (left ventricular ejection fraction), systematic review, transthoracic echocardiogram

## Abstract

Left ventricular ejection fraction (LVEF) guides diagnosis and treatment across cardiovascular disease, but its measurement from transthoracic echocardiography (TTE) is operator-dependent and subject to observer variability. Artificial intelligence (AI) methods that estimate a continuous LVEF value have proliferated. This systematic review aimed to summarise how closely they agree with reference measurements at the group and individual-patient level.

Following PRISMA 2020 guidelines and protocol registration on the International Prospective Register of Systematic Reviews (PROSPERO: CRD420261419064), we searched MEDLINE and Embase (via Ovid), Scopus, and IEEE Xplore from January 2015 to 10 September 2026, with forward and backward citation searching, for peer-reviewed journal articles of AI-derived continuous LVEF from standard adult TTE that reported an error or agreement metric against a reference standard. Risk of bias was assessed with a tailored Quality Assessment of Diagnostic Accuracy Studies 2 (QUADAS-2) tool. Results were summarised as unweighted study-level medians without pooling.

We included 108 studies. Most were retrospective (86 of 108), and 34 reported an external or independent test cohort as their main result. EchoNet-Dynamic or CAMUS were used in 54 studies. Systematic bias was small (median absolute bias: 1.5 EF percentage points, interquartile range: 0.5 to 3.4; 68 studies). Typical average error was moderate (median mean absolute error: 4.9 points, 62 studies). The median width of the study-specific 95% limits of agreement was 26 points (interquartile range: 22 to 30; 66 studies). Only 10 of 66 studies (15%) had limits within ±10 points, the usual gap between clinical LVEF decision thresholds. The limits were similarly wide when external results were used wherever available (median width: 26.3 points) and somewhat narrower in studies at low risk of bias (23.6 points).

AI methods reproduce reference LVEF with small systematic bias, but individual-patient disagreement in most studies exceeds the difference between clinical LVEF categories and the agreement expected between two expert readers. On current evidence, automated LVEF is better suited to assistive use with expert oversight than to stand-alone measurement, pending prospective, externally validated evaluation.

## Introduction and background

Left ventricular ejection fraction (LVEF), the proportion of end-diastolic volume ejected with each beat, is the most widely used quantitative measure of left ventricular systolic function. It informs the classification and management of heart failure, prognostic assessment, treatment monitoring, and selection for pharmacological and device therapies [1].

Standard transthoracic echocardiography (TTE) is the first-line method for assessing left ventricular systolic function and the setting in which LVEF is most often measured. For two-dimensional TTE, current recommendations favour the biplane method of disks from the apical four- and two-chamber views when image quality permits [2]. This requires view selection, identification of end-diastolic and end-systolic frames, and manual tracing of the endocardial border, so it is time-consuming and affected by image quality and operator judgement. Visual estimation is faster but depends on experience, and both quantitative and visual assessment are subject to intra-observer, inter-observer, and test-retest variability [2,3].

Artificial intelligence (AI) methods can now estimate LVEF automatically from echocardiographic images and video, either by copying the manual workflow (finding the views and frames, tracing the ventricle, and calculating volumes) or by predicting LVEF directly [4]. The clinical question is whether these methods can accurately automate or replace the manual measurement workflow, which depends on how closely AI estimates agree with an established reference measurement in individual patients [5,6]. We therefore restricted this review to AI methods producing a continuous LVEF value, firstly because only a continuous value allows agreement to be quantified in ejection fraction (EF) percentage points and compared with known inter-observer variability [3], and secondly because clinical decisions, for example in heart failure management and monitoring during cancer therapy, depend on specific thresholds and on serial change [1], which categorical outputs capture only partially.

Agreement is described by three measures: the mean absolute error (MAE), the average size of the difference; the Bland-Altman bias, the systematic tendency to over- or underestimate; and the 95% limits of agreement (LoA), the range within which most individual differences fall. Studies differ in models, datasets, reference standards [7], and which of these measures they report [4], which limits comparison between them. A synthesis of individual-level agreement against clinically meaningful limits, by validation level and risk of bias, is lacking in the literature.

This review aims to characterise AI methods that produce a continuous LVEF value from standard adult TTE, summarise their reported error and agreement with clinical reference standards, and assess the risk of bias and applicability of the available evidence.

This article was previously presented as a meeting abstract at the International Conference on Artificial Intelligence in Healthcare 2026 (AIiH 2026), London, on 27 August 2026.

## Review

Methods

Protocol and Registration

This review is reported according to the Preferred Reporting Items for Systematic Reviews and Meta-Analyses (PRISMA) 2020 statement [8] and its search extension PRISMA-S [9], and was registered with the International Prospective Register of Systematic Reviews (PROSPERO: CRD420261419064) on 17 June 2026 and later amended.

Eligibility Criteria

Studies were included if they: (1) enrolled adults aged 18 years or older undergoing standard TTE for any clinical indication, with public standard-TTE datasets such as EchoNet-Dynamic [10] and CAMUS [11] eligible where the reference standard qualified; (2) evaluated an AI method, of any machine-learning or deep-learning architecture or pipeline, that produced a continuous LVEF value from standard TTE images or video; (3) compared it with an established reference standard: expert manual echocardiographic measurement (Simpson biplane or monoplane, or visual estimation by a trained echocardiographer), core laboratory measurement, cardiac magnetic resonance (CMR) imaging, three-dimensional echocardiography, semi-automated echocardiographic software, or public-dataset labels derived from one of these, recognising that these references are related but not interchangeable [7]; (4) reported at least one agreement or error metric (MAE, root mean squared error (RMSE), Bland-Altman bias or limits of agreement); and (5) were full-text, peer-reviewed journal articles in English published from 1 January 2015.

Studies were excluded if they: (1) used point-of-care, bedside, focused, or handheld ultrasound as the primary modality, because operators, equipment, and image quality differ from standard TTE; (2) used a non-echocardiographic input to the AI model, or exclusively three-dimensional echocardiographic input; (3) reported only segmentation accuracy, view classification, image quality, disease detection, or right ventricular function, with no continuous LVEF agreement; or (4) were reviews, editorials, letters, commentaries, conference abstracts, conference proceedings, or preprints.

A method was considered automated if it computed LVEF without manual tracing or editing of the endocardial border. Operator selection of the view, loop, or end-diastolic and end-systolic frames, or their provision with a public dataset, was permitted as part of the standard clinical workflow.

The primary outcomes were the MAE, RMSE, Bland-Altman bias (mean difference), and Bland-Altman 95% LoA, all expressed in EF percentage points [5,6]. Secondary outcomes, extracted where reported, were technical success (the proportion of examinations successfully analysed), the intraclass correlation coefficient (ICC), coefficient of determination (R²), and Pearson or Spearman correlation coefficient [5,12].

Information Sources and Search Strategy

MEDLINE ALL and Embase (both via Ovid), Scopus, and IEEE Xplore were searched from 1 January 2015 to 10 September 2026. The search combined free-text terms for artificial intelligence and machine learning, echocardiography, and LVEF in titles and abstracts (MEDLINE and Embase), titles, abstracts, and keywords (Scopus), and all metadata fields (IEEE Xplore). Searches were limited by publication year, and in Scopus and IEEE Xplore to journal articles and conference papers, with eligibility for publication type further applied at screening. Forward and backward citation searching was performed with citationchaser (Lens.org) [13] and updated to 2 October 2026, using the 82 studies included from the database searches as seeds. Full strategies, limits, run dates, and yields for each source are provided in Supplementary Material S1.

Study Selection

Records were de-duplicated with the Ovid de-duplication function and then across databases by digital object identifier and title, with borderline pairs reviewed manually. Records that were not primary research by their recorded publication type (conference abstracts, preprints, trial registrations, errata) were removed before screening. Decisions on records from the original search, screened in Rayyan (Rayyan, Cambridge, MA) [14], were carried forward. Two reviewers independently screened the remaining records against the eligibility criteria, first by title and abstract and then by full text, and disagreements were resolved by consensus. Records from citation searching were de-duplicated against each other and against the database records and screened in the same way. Reasons for full-text exclusion were recorded for each report and are listed in Supplementary Material S1.

Data Collection and Data Items

Two reviewers independently extracted data from each study using a standardised spreadsheet, with disagreements resolved by consensus. The form captured study, population, model, and reference-standard characteristics, the level of human input, the test set and unit of analysis, technical success, reasons for AI failure, pre-analysis exclusions for image quality or missing views, and all agreement and error metrics. Every variable is listed in Supplementary Material S3. All eligible models and test cohorts were extracted, and each cohort was classified as cross-validation (performance averaged across repeated train-test splits of the development data, without a separate test set), internal, or external. A cohort was classified as external if it came from an institution or dataset not used for model development, including fine-tuning, or if a commercial or pre-trained tool was applied to it without retraining. Held-out test sets from the development data and separately collected cohorts from the development institution or dataset, including later or prospective cohorts (temporal validation), were classified as internal.

For the study-level summaries, one headline result per study was selected in a fixed order that formalised the rule used at data extraction (primary model and main test cohort): (1) the model presented by the authors as primary; (2) where that model was compared with more than one reference standard, the comparison presented as primary; (3) the internal held-out test cohort; (4) where none existed, the cross-validation estimate; and (5) otherwise the first eligible cohort reported, with patient-level results preferred to examination- or video-level results. Each study was counted once. For sensitivity analyses, the external results of each study were reduced to one value, the median of its external results against the same reference modality as its headline result (echocardiography or CMR), so that several cohorts from one study were not treated as independent. These values were summarised across studies, overall and separately for independent clinical and public datasets, used in place of the headline result wherever available, and compared with the headline result within studies. All eligible results of each study (internal, cross-validation, and external) were also reduced to one value in the same way and summarised across all studies.

All values were recorded in EF percentage points. Where not reported directly, the standard deviation of the differences was derived as (upper LoA - lower LoA)/3.92, and the LoA as bias ± 1.96 standard deviations. Values available only in figures were read manually from the enlarged figures against the axis scale. Derived and figure-read values were labelled. Bias was recorded as AI minus reference where the direction could be established, but because the direction was not reported consistently, synthesis relied on absolute bias. Values that were internally inconsistent in the source paper were checked against the full text and flagged. The extraction dataset, with the source and provenance of each value, is provided in Supplementary Material S3.

Risk of Bias

Risk of bias and applicability were assessed with the Quality Assessment of Diagnostic Accuracy Studies 2 (QUADAS-2) tool [15], tailored in advance for an AI index test and a continuous agreement outcome. As a continuous LVEF output has no diagnostic threshold, the threshold question in the index test domain was replaced with one on data leakage: whether the test set was independent, at patient level, of the data used to develop the model. In the reference standard domain, each reference was judged on the rigour of the method described rather than by reference type. The form was piloted on five studies, and two reviewers assessed each study independently, with disagreements resolved by consensus. Supplementary Material S2 gives the full form, decision rules, and study-level judgements, including how participant overlap, overfitting, reference-reader blinding, and technical failures were judged.

Synthesis of Results

No meta-analysis was undertaken [16] due to heterogeneity of results. Random-effects pooling of Bland-Altman limits of agreement [17] was considered but not used, because AI methods, units of analysis (patients, examinations or video clips), reference standards, and populations differed, agreement statistics were often incomplete, and several studies reused the same datasets.

A structured synthesis without meta-analysis was performed following the Synthesis Without Meta-analysis (SWiM) guideline [18]. Results were summarised as unweighted study-level medians, interquartile ranges (IQR), and ranges with explicit denominators. These describe the distribution of study results and are not pooled estimates of performance, and no inferential statistics were calculated. Likewise, the medians of the study-specific lower and upper LoA describe typical studies and do not form a pooled interval. Absolute bias and LoA width (upper minus lower limit) were summarised alongside signed values. Approximate 95% confidence intervals for each study-specific LoA were calculated from the reported sample size [19]. For examination- and video-level results, which may include more than one observation per patient, these intervals assume independent observations and are therefore likely to be too narrow. We counted the studies whose LoA met a clinical reference of ±10 EF percentage points (defined as a total LoA width of 20 points or less). This reference was chosen because clinical decisions use LVEF thresholds about 10 points apart [20,21] and repeat manual two-dimensional measurements by experienced readers typically agree within about ±10 points [22,23], so the reference reflects clinical use for individual patients. The analysis code that reproduces the study-level summaries, sensitivity analyses, and figures is provided in Supplementary Material S4.

Results were stratified by validation level, reference standard, model type (commercial pre-trained or developed in the study), AI approach, input type, and study design. Sensitivity analyses were restricted to studies at low risk of bias in all four domains, to external validation cohorts, to studies at low risk of bias with external validation, to fully automated pipelines without human selection, to directly reported values, to patient-level results, to institutional datasets and to studies without a data-reliability flag. The relation between test cohort size and agreement was examined with the Spearman correlation coefficient.

Amendments to the Protocol

The review departed from the registration in three ways. The clinical setting was described too inconsistently to analyse, so the data source was examined instead, in a sensitivity analysis restricted to institutional datasets. The sign convention for bias also varied, so the planned vote count on its direction was replaced by a summary of absolute bias. Finally, QUADAS-2 was tailored as aforementioned.

Further changes were made in response to peer review. The searches were updated to 10 September 2026 and extended to IEEE Xplore, with publication-type limits applied at screening, and backward citation searching was carried out and forward citation searching added, both run on 28 September and updated to 2 October 2026. Extraction was widened to all eligible cohorts, to the ICC, R², correlation coefficients, and technical success as secondary outcomes, and to reasons for AI failure and exclusions for image quality or missing views. The order for selecting the headline result was formalised, and the additional analyses described above were performed. Eligibility criteria were stated in more operational detail without changing their scope, and the restriction to peer-reviewed journal articles was retained. All amendments were recorded in PROSPERO on 12 October 2026.

Results

Study Selection

The database searches identified 3,675 records (MEDLINE and Embase via Ovid = 1,920; Scopus = 1,419; IEEE Xplore = 336) in the update of the original search. After removal of 1,193 duplicates and 693 records ineligible by publication type, 1,789 records were screened, 132 reports were sought, 129 were assessed in full, and 82 studies were included. Citation searching identified 6,670 records, from which 50 reports were assessed in full, and 26 further studies were included. In total, 108 studies were included [10,11,24-129]. Reports excluded at full text commonly included evaluations of automated LVEF using point-of-care or handheld ultrasound [130,131], three-dimensional input [132], or a paediatric population [133], or with no continuous agreement metric (Figure 1) [134].

**Figure 1:**
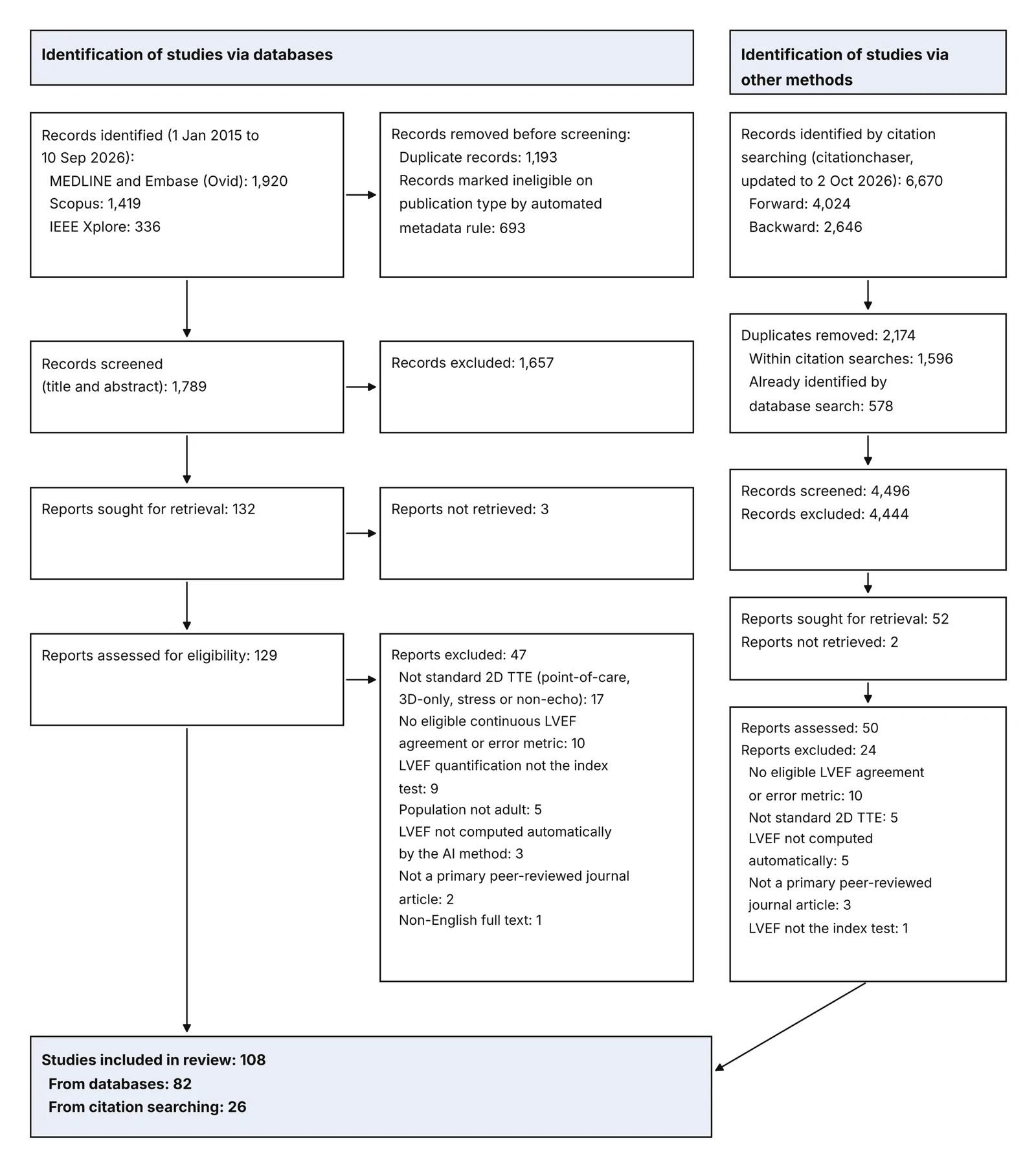
PRISMA 2020 flow diagram of study selection. Records identified through databases and through citation searching are shown separately; reasons for full-text exclusion are grouped into categories. TTE, transthoracic echocardiography; LVEF, left ventricular ejection fraction.

Study Characteristics

Characteristics of the included studies are summarised in Table 1 and given for each study in Supplementary Material S3. Test cohort size ranged from 6 to 100,994 units (median: 500), and where a cohort mean LVEF was reported (48 studies), it was usually in the normal range (median: 55.4%).

**Table 1:** Characteristics of the 108 included studies. Values are the number of studies (%). AI, artificial intelligence; CMR, cardiac magnetic resonance; MAE, mean absolute error. Categories within a section are mutually exclusive, except for rows marked "any" and single-characteristic rows such as video input and the Reporting rows. Country is that of the data source of the headline result, first listed where there were several, and public datasets are counted by their country of origin (USA for EchoNet-Dynamic, France for CAMUS). Study-level details are given in Supplementary Material S3.

Characteristic	Studies, n (%)
Publication year	
2015-2021	15 (14)
2022-2026	93 (86)
Country of data source (first listed)	
USA	44 (41)
China	11 (10)
France	18 (17)
Norway	6 (6)
Japan	4 (4)
Other or multinational	25 (23)
Design	
Retrospective only	86 (80)
Prospective	11 (10)
Retrospective development with prospective component	11 (10)
Data	
EchoNet-Dynamic used (any cohort)	36 (33)
CAMUS used (any cohort)	29 (27)
Public benchmark data only	37 (34)
Any institutional cohort	71 (66)
Validation of headline result	
Internal held-out test set	63 (58)
Cross-validation without separate test set	11 (10)
External or independent cohort	34 (31)
Any external cohort reported	58 (54)
AI method	
Commercial product applied without retraining	29 (27)
Developed in study: segmentation/measurement-then-volume	49 (45)
Developed in study: end-to-end regression	28 (26)
Developed in study: landmark-based or other	2 (2)
Video input	87 (81)
No human selection of view, loop or frames	80 (74)
Reference standard of headline result	
Expert echocardiography	44 (41)
Public-dataset labels	42 (39)
Routine clinical report	16 (15)
Cardiac magnetic resonance	4 (4)
Three-dimensional echocardiography	2 (2)
Any comparison with CMR	7 (6)
Unit of analysis of headline result	
Patient	66 (61)
Examination	17 (16)
Video clip	25 (23)
Reporting	
Technical success reported	27 (25)
Agreement stated as complete-case	30 (28)
MAE reported	62 (57)
Bland-Altman limits of agreement reported or derivable	66 (61)

Among the 79 models developed in the studies, convolutional networks predominated (51, including six combined with recurrent networks), with transformer or hybrid architectures in 18 and foundation or vision-language models in six. Where reported, technical success had a median of 96.0% (range: 51% to 100%) and was below 90% in nine studies. Reasons for AI failure, reported in 17 studies, were most often poor image quality or missing or uninterpretable apical views, and 28 studies excluded examinations for image quality or missing views before analysis.

The headline result came from an internal held-out test set in 63 studies, from cross-validation without a separate test set in 11, and from an external or independent cohort in 34, of which 29 were commercial tools; 58 studies reported at least one external cohort. The reference standard for the headline result was expert echocardiographic measurement in 44 studies, public-dataset labels in 42, routine clinical reports in 16, CMR in four and three-dimensional echocardiography in two; seven studies reported a comparison with CMR in any cohort.

Agreement and Error

Study-level results are summarised in Table 2 and shown for each study in Figure 2 and Supplementary Material S5. The median MAE was 4.9 EF percentage points (IQR: 4.0 to 5.7; range: 1.6 to 10.8; 62 studies) and the median RMSE was 6.5 (IQR: 5.2 to 7.7; 33 studies). Systematic bias was small in most studies: the median absolute bias was 1.5 points (IQR: 0.5 to 3.4; range: 0.0 to 5.8; 68 studies), and five studies had an absolute bias above five points. Where its direction could be established (67 studies), the AI value was lower than the reference in 23, equal in two, and higher in 42, and the median signed bias was 0.3 points.

**Table 2:** Error and agreement by subgroup and in sensitivity analyses (headline result per study). Values are unweighted study-level medians (interquartile range); n = number of studies contributing, in EF percentage points. They describe the distribution of study results and are not pooled estimates. Absolute bias is the absolute value of the reported Bland-Altman mean difference; LoA width is the upper minus the lower 95% limit of agreement. MAE, mean absolute error; LoA, limits of agreement; EF, ejection fraction.

Group	Studies	MAE	Absolute bias	LoA width
All studies				
All included studies	108	4.9 (4.0-5.7); n=62	1.5 (0.5-3.4); n=68	26.0 (21.6-30.1); n=66
Validation level of headline result				
Internal held-out test	63	4.8 (4.0-5.7); n=47	1.2 (0.4-3.6); n=27	25.0 (20.9-28.8); n=27
Cross-validation only	11	5.3 (5.1-6.1); n=7	1.5 (0.9-2.8); n=10	25.5 (21.9-27.9); n=10
External or independent cohort	34	4.9 (4.3-6.8); n=8	1.5 (0.6-3.3); n=31	28.8 (22.4-31.8); n=29
Reference standard of headline result				
Expert echocardiography	44	5.1 (4.2-5.7); n=16	1.7 (0.9-4.4); n=36	24.6 (21.6-29.8); n=34
Public-dataset labels	42	5.0 (4.0-5.7); n=35	1.1 (0.7-2.6); n=18	25.8 (21.9-28.0); n=18
Routine clinical report	16	4.8 (4.2-7.0); n=9	0.2 (0.2-3.0); n=9	29.2 (22.1-40.0); n=9
Cardiac magnetic resonance	4	-	1.9 (1.1-2.6); n=4	30.2 (27.8-32.8); n=4
Three-dimensional echocardiography	2	3.5 (3.0-4.0); n=2	0.3 (0.3-0.3); n=1	11.9 (11.9-11.9); n=1
Model type and approach				
Commercial, applied without retraining	29	5.1 (4.5-6.7); n=5	1.6 (0.5-3.5); n=26	27.6 (22.9-31.8); n=24
Developed in study: segmentation/measurement-then-volume	49	5.1 (4.4-5.7); n=29	1.5 (0.9-3.5); n=35	26.0 (21.2-29.7); n=35
Developed in study: end-to-end regression	28	4.4 (4.0-5.5); n=26	0.2 (0.2-0.9); n=7	22.1 (20.7-26.5); n=7
Study design				
Retrospective	86	4.8 (4.0-5.7); n=56	1.0 (0.4-2.9); n=51	25.8 (21.7-31.2); n=52
Prospective	11	7.4 (7.4-7.4); n=1	4.3 (3.0-4.8); n=9	26.3 (24.2-29.1); n=7
Retrospective development with prospective component	11	5.5 (3.7-5.6); n=5	1.4 (0.2-2.8); n=8	25.5 (20.9-27.0); n=7
Sensitivity analyses				
Low risk of bias in all four domains	17	4.5 (3.4-5.1); n=4	3.0 (1.9-4.6); n=15	23.6 (22.4-26.7); n=13
Low risk of bias and external validation	9	-	3.0 (2.2-4.3); n=9	23.6 (22.4-26.7); n=8
External results used wherever available	108	5.1 (4.4-6.1); n=62	1.5 (0.6-3.4); n=70	26.3 (21.6-30.0); n=67
External results only, one value per study	58	5.4 (4.7-6.6); n=26	1.5 (0.7-3.3); n=41	26.3 (22.4-31.6); n=39
All results, one value per study	108	5.0 (4.4-6.1); n=62	1.5 (0.7-3.2); n=71	26.5 (21.7-30.5); n=68
Fully automated (no human selection)	80	4.6 (4.0-5.8); n=44	1.6 (0.5-3.6); n=49	25.5 (21.5-29.7); n=47
Directly reported values only	56	5.0 (4.0-5.7); n=42	1.5 (0.9-3.9); n=22	23.9 (22.0-27.4); n=19
Patient-level headline results only	66	5.0 (4.2-5.6); n=31	1.5 (0.6-3.7); n=51	26.5 (21.7-31.1); n=48
Institutional data only (no public dataset in headline)	66	4.8 (4.1-6.2); n=27	1.5 (0.5-3.7); n=50	26.0 (21.7-31.5); n=48
Excluding studies with a data-reliability flag	99	5.0 (4.1-5.7); n=57	1.4 (0.4-3.0); n=62	25.3 (21.5-29.9); n=60

**Figure 2:**
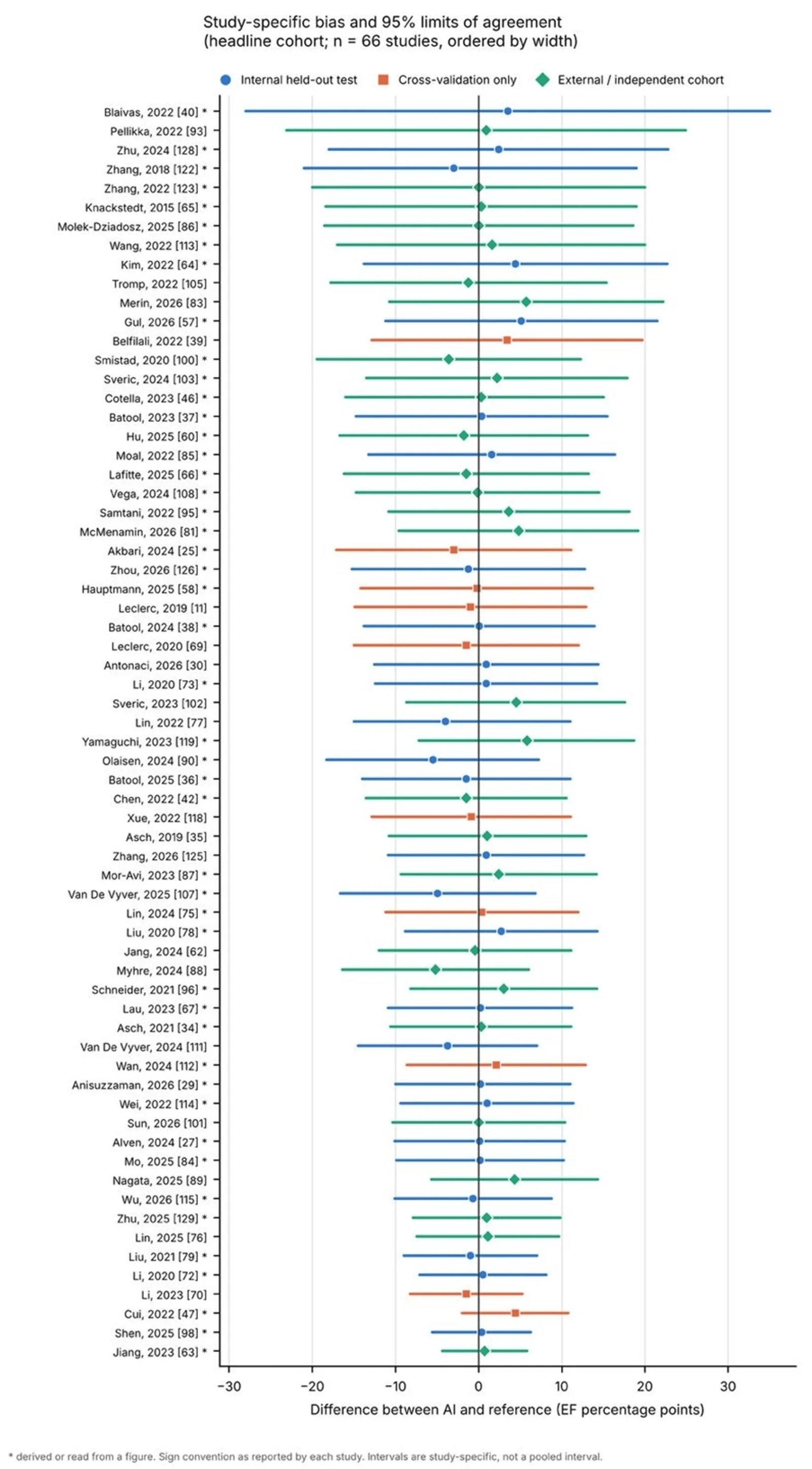
Study-specific Bland-Altman bias and 95% limits of agreement for the headline result of each study reporting limits of agreement (n = 66), ordered by the width of the interval. Bias is shown with the sign reported by each study. The intervals are study-specific and are not pooled. Marker shape and colour show the validation level. * marks values derived from reported statistics or read from figures.

Individual-level agreement was wider. The median width of the study-specific 95% LoA was 26.0 points (IQR: 21.6 to 30.1; range: 10.2 to 63.0; 66 studies), with median study-specific lower and upper limits of -12.7 and +12.9 points. These are medians of separate study-specific intervals, not a pooled interval. The LoA estimates rested on 89,709 analysis units (median: 429 per study), and approximate confidence intervals, calculable for the 62 studies that reported a sample size, are given in Supplementary Material S3. In the 27 studies that reported both, LoA width was a near-constant multiple of MAE (median ratio: 4.93, IQR: 4.73 to 5.28). Secondary measures were reported inconsistently: median Pearson r = 0.85 (57 studies), R² = 0.81 (21) and ICC = 0.81 (18).

Ten of 66 studies (15%) had 95% LoA within ±10 points, including four of 29 external validation cohorts and one of 13 studies at low risk of bias in all domains.

Subgroups and Sensitivity Analyses

Results by subgroup and sensitivity analyses are shown in Table 2, and all comparisons are descriptive. External headline results had a similar MAE to internal ones but wider LoA, and most (29 of 34) came from commercial tools. Among external results, the median MAE was 5.0 for independent clinical datasets (22 studies) and 6.1 for public datasets (nine studies). In the 18 studies reporting MAE for both an internal or cross-validated result and an external result, MAE was higher externally in 14 (median difference: 0.6 points), whereas LoA width differed little (median difference: −1.2 points, nine studies).

Commercial tools, most tested on independent clinical cohorts, had wider LoA than models developed in the studies. Studies at low risk of bias in all four domains had narrower LoA but larger absolute bias, and prospective studies also had larger absolute bias.

Against CMR (seven studies, eight cohorts), the median LoA width was 30.2 points, compared with 24.6 against expert echocardiography. Six of these seven studies evaluated commercial tools, mostly in single-centre cohorts of 48 to 395 patients with specific indications such as heart failure, cardiomyopathy, or cancer therapy, and absolute bias reached 11.0 points.

Restricting to fully automated pipelines, directly reported values, patient-level results, or studies without a data-reliability flag changed the median MAE by no more than 0.4 points and the median LoA width by no more than 2.1 points. Error was not related to test cohort size (Spearman correlation with LoA width of 0.11, 62 studies; with MAE of 0.03, 57 studies).

End-to-end models had a slightly lower median MAE than segmentation- or measurement-then-volume pipelines (4.4, 26 studies, versus 5.1, 29 studies), and methods analysing video had a lower median MAE than those analysing still frames (4.7, 46 studies, versus 5.5, 16 studies). Both comparisons are confounded by dataset and validation design, because most end-to-end results came from internal test sets of public datasets and 12 of the 16 frame-based results came from public datasets, mostly CAMUS.

Risk of Bias

Risk of bias and applicability are summarised by domain in Table 3, with study-level judgements and rationale in Supplementary Material S2. Seventeen of the 108 studies were at low risk of bias in all four domains and 32 at high risk in at least one. High risk arose mainly from selected or enriched samples (patient selection), cross-validation without a held-out test set or overlap between test and development data (index test), and exclusion of cases the AI could not analyse (flow and timing). Reference standard ratings were most often unclear because the method or readers behind routine clinical or public-dataset labels were not reported. Applicability concerns were high in 18 studies, mostly because of narrow single-disease populations. Agreement in studies at low risk of bias is reported with the sensitivity analyses in Table 2.

**Table 3:** Risk of bias and applicability (tailored QUADAS-2). Number of studies = 108. Full judgements, signalling questions, and supporting rationale for each study are given in Supplementary Material S2. Applicability was rated for the first three domains only. QUADAS-2, Quality Assessment of Diagnostic Accuracy Studies 2.

Domain	Low	High	Unclear
Patient selection: risk of bias	73	15	20
Patient selection: applicability	88	17	3
Index test: risk of bias	85	13	10
Index test: applicability	106	2	0
Reference standard: risk of bias	54	4	50
Reference standard: applicability	106	2	0
Flow and timing: risk of bias	65	8	35

Discussion

Principal Findings

In this systematic review of 108 studies, AI methods estimated LVEF from standard adult TTE with small systematic bias in most studies (median absolute bias: 1.5 EF percentage points) and a median MAE of about five points. Individual-level agreement was wider: the median width of the study-specific 95% LoA was 26 points. Only 15% of studies had limits within ±10 points, and LoA widths were similar when external cohorts were used wherever available and somewhat narrower in studies at low risk of bias (23.6 points), although their absolute bias was larger (3.0 points).

Interpretation

These results indicate that current AI methods estimate LVEF reasonably well on average but not yet closely enough in individual patients to replace expert measurement. The three measures answer different questions: the MAE gives the typical size of error, bias whether a method over- or underestimates, and the LoA how far an individual result may fall from the reference. Absolute bias was preferred to the median signed bias (0.3 points), which is near zero because positive and negative biases cancel across studies and does not show that any individual method is unbiased.

On current evidence, AI cannot yet replace expert LVEF measurement, because individual-level agreement is too wide. Judged against a ±10-point reference, the individual-level disagreement in most studies was large enough to move a patient across the thresholds that separate reduced, mildly reduced, and preserved ejection fraction [20], or to mimic or mask a 10-point change used to define cancer therapy-related dysfunction [21]. It was also wider, in most studies, than the agreement usually reported between repeat expert measurements [22,23]. Agreement between an AI method and a single reference measurement combines error from the AI, the reference reader, acquisition, and biological variation. These components cannot be separated with study-level data, and the share attributable to the reference cannot be estimated here. The wider limits against CMR are consistent with the known differences between echocardiographic and CMR volumes [7], rather than evidence that AI performs worse against CMR.

These findings are consistent with a recent meta-analysis of AI and clinician LVEF estimates, which reported similar limits of agreement (about ±13 points) [135], and with a review of AI-assisted point-of-care ultrasound, in which limits were wider [136]. The present review adds a larger set of standard TTE studies, analysis by validation level, reference standard and risk of bias, and interpretation against a clinical reference.

These findings support the use of automated LVEF as an assistive measurement reviewed by an expert, for example to provide a first estimate, reduce workload, or flag discordant results, rather than as a stand-alone replacement for expert measurement where a single value informs a decision near a threshold, such as device eligibility or monitoring during cardiotoxic therapy [1,21]. This conclusion applies to the published evidence as a whole, not to every tool, and some individual tools had limits within ±10 points.

Limitations of the Evidence

The reference standard was almost always echocardiography, often a single value taken from a clinical report or public dataset without detail of how it was measured, so the results show how closely AI reproduces echocardiographic measurements rather than its accuracy against CMR [7], which only seven studies used. Most studies were retrospective, and EchoNet-Dynamic and CAMUS were used in half of them, so the 108 studies are not independent populations. Each study was counted once, and no cohort contributed more than once to any summary, but the summaries do not adjust for studies sharing the same datasets. Units of analysis also differed between patients, examinations, and video clips. Technical success was reported in only a quarter of studies, and agreement was often calculated only in cases both methods could analyse, which may overstate agreement in routine practice. Few studies reported agreement separately by LVEF range, rhythm, regional wall motion, or image quality, so performance in reduced LVEF, arrhythmia, and technically difficult examinations could not be summarised, and reported cohort means were usually in the normal range.

Limitations of This Review

The search used free-text terms only, and peer-reviewed conference proceedings were excluded because they often lack the detail needed for risk of bias assessment and many are later published as journal articles, so some technical evaluations may have been missed and the findings apply to peer-reviewed journal evidence. The hierarchy for selecting each study's headline result was not prespecified, although sensitivity analyses using external or all results gave similar findings, and later cohorts from the development institution (temporal validation) were counted as internal rather than analysed separately. Heterogeneity precluded meta-analysis [16], and the unweighted medians give small and large studies equal weight, although error was not related to test cohort size. Some values were derived or read from figures, and the direction of bias could not be harmonised for every study.

Implications for Research

The main gap is prospective, multicentre evaluation in unselected consecutive examinations. Such studies should use a rigorous reference, such as blinded multi-reader measurement or CMR, and compare AI-human with human-human agreement in the same cohort to show how much disagreement reflects the reference. They should report technical success and the handling of unanalysable cases, the unit of analysis, and Bland-Altman bias and LoA with confidence intervals across the range of LVEF. Testing several AI methods on the same patients would show which perform better, independent of differences between datasets.

## Conclusions

Automated AI methods estimated LVEF from standard adult echocardiography with small systematic bias and moderate average error in peer-reviewed journal studies. In most studies, the limits of agreement for individual patients were wider than the spacing of clinical LVEF thresholds and the agreement expected between expert readers. These findings describe the published evidence as a whole, not every tool. On current evidence, automated LVEF is better suited to assistive use with expert oversight than to stand-alone measurement. Prospective, externally validated studies reporting individual-level agreement against a rigorous reference are needed to establish where it can assist or reduce the need for manual measurement, and substitution for expert assessment would also require prospective evidence of technical failure rates in routine examinations and of its effect on clinical decisions.

## Figures and Tables

**Figure 3:**
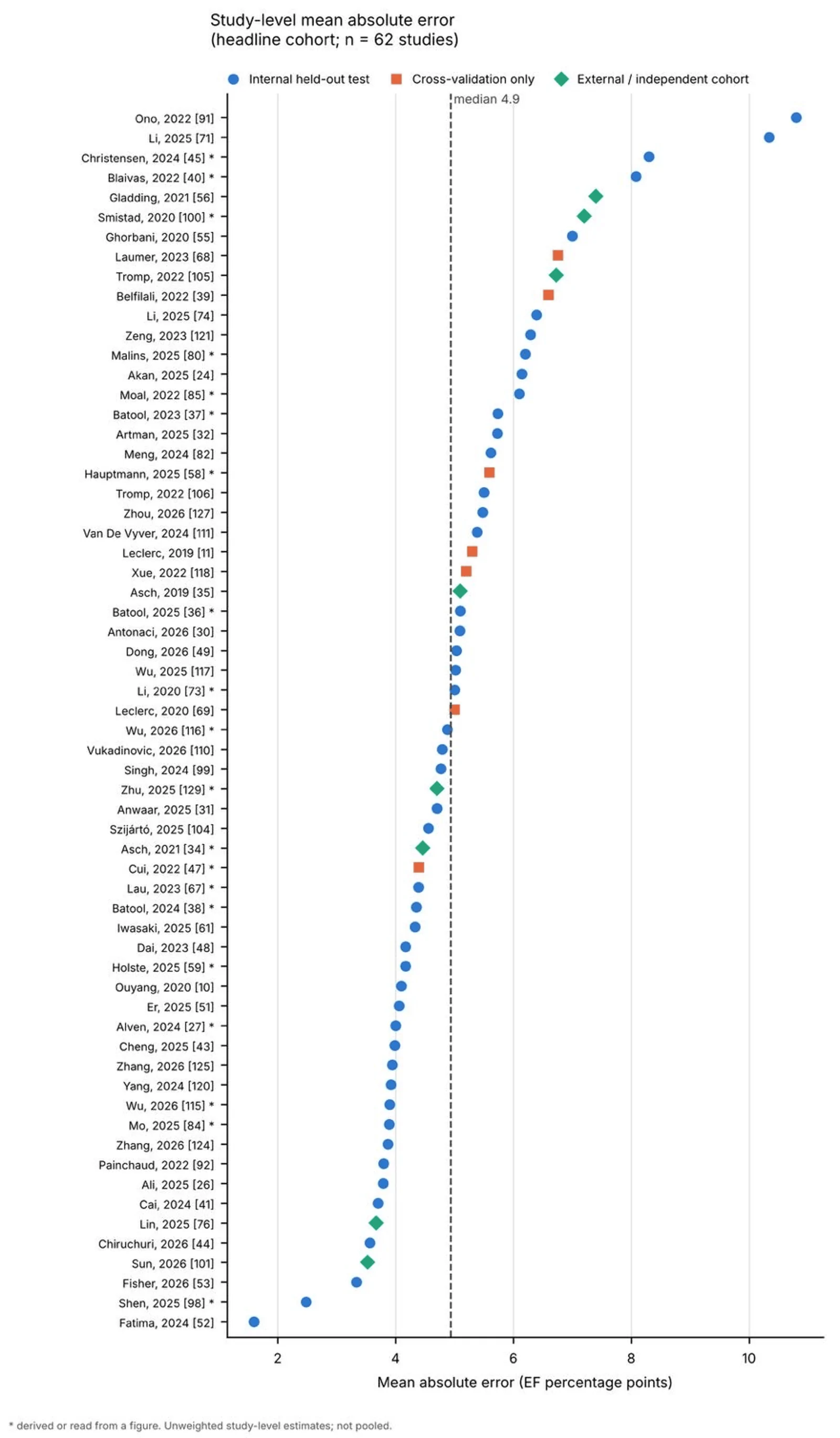
Study-level mean absolute error for the headline result of each study reporting MAE (n = 62), ordered by value. The dashed line marks the median. Marker shape and colour show the validation level. * marks values derived from reported statistics or read from figures. Values are unweighted and not pooled. MAE, mean absolute error.

## Appendices

Supplementary material

Artificial intelligence for automated continuous quantification of left ventricular ejection fraction from standard adult transthoracic echocardiography: a systematic review

Contents

Supplementary Material S1. Search strategies, limits, run dates, and yields for each source (PRISMA-S)

Supplementary Material S2. Risk of bias and applicability assessment (tailored QUADAS-2): form, decision rules, and study-level judgements

Supplementary Material S3. Extraction dataset 

Supplementary Material S4. Analysis code 

Supplementary Material S5. Study-level mean absolute error

Supplementary Material S1. Search strategies, limits, run dates, and yields for each source (PRISMA-S)

All databases were searched from 1 January 2015 and last searched on 10 September 2026. This update re-ran the full date window and superseded the initial searches of MEDLINE, Embase, and Scopus run on 30 April 2026; IEEE Xplore was added at the update. No limits other than publication year and the publication-type limits shown were applied, and no language limit was used. The search strategy was not peer-reviewed, and no grey literature, trial registers, or websites were searched. Study authors were not contacted (Table 4).

**Table 4:** Sources, run dates, limits, and records retrieved. Citation searching was run with citationchaser (Lens.org) on 28 September 2026 and updated on 2 October 2026, using the 82 studies included from the database searches as seeds.

Source	Platform	Coverage/Seeds	Last searched	Limits	Records
MEDLINE ALL	Ovid	1946 to September 2026	10 Sep 2026	Publication year 2015 onwards	1.920 combined with Embase (1,399 after Ovid de-duplication)
Embase	Ovid	1974 to September 2026	10 Sep 2026	Publication year 2015 onwards	(Combined with MEDLINE, above)
Scopus	Elsevier	All years	10 Sep 2026	Document type: article or conference paper; publication year 2015 to 2026	1,419
IEEE Xplore	IEEE	All years	10 Sep 2026	Journals and conferences; 2015 to 2026	336
Forward citation searching	citationchaser (Lens.org)	Citing articles of the 82 studies included from the database searches	2 Oct 2026	None	4,024
Backward citation searching	citationchaser (Lens.org)	References of the 82 studies included from the database searches	2 Oct 2026	None	2,646

Ovid MEDLINE ALL and Embase (searched together; fields, ti,ab.)

(1) (artificial intelligence or machine learning or deep learning or neural network* or convolutional neural network* or CNN).ti,ab. {963,770}.

(2) (echocardiograph* or echocardiogram* or cardiac ultrasound or transthoracic or TTE or echo).ti,ab. {768,090}.

(3) (ejection fraction or LVEF or left ventricular ejection fraction or systolic function or ventricular function).ti,ab. {416,003}.

(4) 1 and 2 and 3 {1,935}.

(5) limit 4 to yr="2015 -Current" {1,920}.

(6) Remove duplicates from 5 {1,399}.

Scopus

(TITLE-ABS-KEY("artificial intelligence" OR "machine learning" OR "deep learning" OR "neural network*" OR "convolutional neural network*" OR "CNN")) AND (TITLE-ABS-KEY("echocardiograph*" OR "echocardiogram*" OR "cardiac ultrasound" OR "transthoracic" OR "TTE" OR "echo")) AND (TITLE-ABS-KEY("ejection fraction" OR "LVEF" OR "left ventricular ejection fraction" OR "systolic function" OR "ventricular function")) AND DOCTYPE(ar OR cp) AND PUBYEAR > 2014 AND PUBYEAR < 2027

IEEE Xplore (Command Search)

(("All Metadata": "artificial intelligence" OR "All Metadata": "machine learning" OR "All Metadata": "deep learning" OR "All Metadata": "neural network*" OR "All Metadata": "convolutional neural network*" OR "All Metadata": CNN) AND ("All Metadata": echocardiograph* OR "All Metadata": echocardiogram* OR "All Metadata": "cardiac ultrasound" OR "All Metadata": transthoracic OR "All Metadata": TTE OR "All Metadata": echo) AND ("All Metadata": "ejection fraction" OR "All Metadata": LVEF OR "All Metadata": "left ventricular ejection fraction" OR "All Metadata": "systolic function" OR "All Metadata": "ventricular function")). Filters: Journals, Conferences; 2015-2026.

De-duplication

Database records were de-duplicated with the Ovid de-duplication function (MEDLINE and Embase; 521 duplicates) and then across databases by exact digital object identifier, exact title, and near-identical title (672 duplicates); 254 borderline pairs were reviewed manually and not merged. Citation-search records were de-duplicated by digital object identifier and normalised title against each other (1,596 duplicates) and against all database records (578 duplicates).

Full-Text Exclusions

Table 5 lists every report excluded after full-text assessment, with the reason agreed by the two reviewers (47 reports from the database searches and 24 from citation searching). The categories match those in Figure 1.

**Table 5:** Reports excluded at full-text assessment, with reasons (n = 71). Categories: Not standard 2D TTE includes point-of-care, handheld, 3D-only, stress, M-mode, and non-echocardiographic input or reference. Reports not retrieved (three from the database searches and two from citation searching) are shown in Figure 1. LVEF, left ventricular ejection fraction; TTE, transthoracic echocardiography; POCUS, point-of-care ultrasound; EF, ejection fraction; LoA, limits of agreement; HUD, handheld ultrasound device; MAE, mean absolute error; MAD, mean absolute deviation; RMSE, root mean square error; CAD, coronary artery disease; DL, deep learning; LV, left ventricular; CHD, congenital heart disease; CMR, cardiac magnetic resonance; EDV, end-diastolic volume; ESV, end-systolic volume; CNN, convolutional neural network; ICC, intraclass correlation coefficient.

Study	Report (title, journal, DOI)	Reason category	Details
Database searches			
Blaivas 2022	Making Artificial Intelligence Lemonade Out of Data Lemons: Adaptation of a Public Apical Echo Database for Creation of a Subxiphoid Visual Estimation Automatic Ejection Fraction Machine Learning Algorithm. Journal of Ultrasound in Medicine: Official Journal of the American Institute of Ultrasound in Medicine. 10.1002/jum.15889	Not standard 2D TTE	POCUS/subxiphoid study; review excludes point-of-care/handheld/focused ultrasound despite continuous EF metric.
Borde 2026	Artificial intelligence-assisted peri-operative echocardiography: a multicentre observational study. Anaesthesia. 10.1111/anae.70235	Not standard 2D TTE	Anaesthetist-acquired 'point-of-care TTE' 12-view limited protocol (device/cart vs. handheld not stated); US2.AI cloud; reference = 'manual measurement by expert clinician'; LVEF method not stated (Simpson vs. visual).
Campbell 2025	Artificial intelligence fully automated analysis of handheld echocardiography in real-world patients with suspected heart failure. European Journal of Heart Failure. 10.1002/ejhf.3783	Not standard 2D TTE	Handheld echocardiography study; primary endpoint is classification of LVEF ≤40%, not standard adult TTE continuous LVEF quantification.
Filipiak-Strzecka 2021	Artificial Intelligence-Powered Measurement of Left Ventricular Ejection Fraction Using a Handheld Ultrasound Device. Ultrasound in Medicine and Biology. 10.1016/j.ultrasmedbio.2020.12.003	Not standard 2D TTE	Handheld ultrasound device study; review excludes handheld/POCUS despite continuous LVEF AI measurement.
Italiano 2021	Feasibility and accuracy of the automated software for dynamic quantification of left ventricular and atrial volumes and function in a large unselected population. Journal of Clinical Medicine. 10.3390/jcm10215030	Not standard 2D TTE	Index test (Philips Dynamic Heart Model) operates on 3D full-volume TTE acquisitions, not standard 2D TTE - a 3D-only index test is an explicit exclusion.
Jafari 2019	Automatic biplane left ventricular ejection fraction estimation with mobile point-of-care ultrasound using multi-task learning and adversarial training. International Journal of Computer Assisted Radiology and Surgery. 10.1007/s11548-019-01954-w	Not standard 2D TTE	Point-of-care ultrasound/mobile POCUS study, which is explicitly excluded despite continuous LVEF estimation.
Kagiyama 2024	Multicenter validation study for automated left ventricular ejection fraction assessment using a handheld ultrasound with artificial intelligence. Scientific Reports. 10.1038/s41598-024-65557-5	Not standard 2D TTE	Handheld AI-POCUS study; excludes point-of-care/handheld ultrasound despite continuous LVEF metrics.
Letizia 2025	Artificial Intelligence Guided Nonexpert Echocardiogram in the COVID-19 Health Action Response in Marines 2.0 Study. Sonography. 10.1002/sono.12541	Not standard 2D TTE	POCUS/AI-guided image acquisition study in mostly very young adults; not standard TTE validation of continuous LVEF against a reference metric; novice operators.
Luong 2024	Validation of machine learning models for estimation of left ventricular ejection fraction on point-of-care ultrasound: insights on features that impact performance. Echo Research and Practice. 10.1186/s44156-024-00043-2	Not standard 2D TTE	POCUS/hand-carried ultrasound study, not standard transthoracic echocardiography.
MacKay 2024	Pragmatic Evaluation of a Deep-Learning Algorithm to Automate Ejection Fraction on Hand-Held, Point-of-Care Echocardiography in a Cardiac Surgical Operating Room. Journal of Cardiothoracic and Vascular Anesthesia. 10.1053/j.jvca.2024.01.005	Not standard 2D TTE	Hand-held point-of-care TTE study (Lumify in OR); POCUS/handheld excluded despite continuous EF metrics.
Morrissey 2026	Assessment of HeartModel R automated left ventricular ejection fraction for patients with hypertrophic cardiomyopathy. European Heart Journal. Imaging Methods and Practice. 10.1093/ehjimp/qyag003	Not standard 2D TTE	Index test is Philips HeartModel run on 3D echocardiographic acquisitions - a 3D-only index test is an explicit exclusion, notwithstanding that the AI output is unedited and real agreement metrics are reported (median difference: -8%, Bland-Altman LoA -23.9.
Motazedian 2023	Diagnostic accuracy of point-of-care ultrasound with artificial intelligence-assisted assessment of left ventricular ejection fraction. npj Digital Medicine. 10.1038/s41746-023-00945-1	Not standard 2D TTE	FoCUS/point-of-care ultrasound study, not standard transthoracic echocardiography input.
Otani 2016	Three-Dimensional Echocardiographic Assessment of Left Heart Chamber Size and Function with Fully Automated Quantification Software in Patients with Atrial Fibrillation. Journal of the American Society of Echocardiography. 10.1016/j.echo.2016.06.010	Not standard 2D TTE	Index test applied to 3D full-volume acquisitions; AI method not described.
Papadopoulou 2022	Clinical validation of an artificial intelligence-Assisted algorithm for automated quantification of left ventricular ejection fraction in real time by a novel handheld ultrasound device. European Heart Journal - Digital Health. 10.1093/ehjdh/ztac001	Not standard 2D TTE	Handheld ultrasound/HUD study; review excludes handheld/POCUS despite continuous AI LVEF metrics.
Papadopoulou 2024	Artificial intelligence-assisted evaluation of cardiac function by oncology staff in chemotherapy patients. European Heart Journal Digital Health. 10.1093/ehjdh/ztae017	Not standard 2D TTE	Handheld ultrasound/HUD study, not standard transthoracic echocardiography per review scope.
Sheikh 2024	Comparing HeartModelAI and cardiac magnetic resonance imaging for left ventricular volume and function evaluation in patients with dilated cardiomyopathy. BMC Cardiovascular Disorders. 10.1186/s12872-024-04355-3	Not standard 2D TTE	Index test is HeartModelAI, which only processes 3D full-volume acquisitions, with no parallel automated 2D-echo arm anywhere in the paper (2D echo appears only as a human-manual enrolment criterion).
Varudo 2022	Machine learning for the real-time assessment of left ventricular ejection fraction in critically ill patients: a bedside evaluation by novices and experts in echocardiography. Critical Care. 10.1186/s13054-022-04269-6	Not standard 2D TTE	Point-of-care/bedside ultrasound as primary modality (paper describes point-of-care echocardiography in ICU patients on a cart-based Venue system); explicit exclusion criterion.
Alam 2022	Ejection Fraction estimation using deep semantic segmentation neural network. Journal of Supercomputing. 10.1007/s11227-022-04642-w	No eligible continuous LVEF agreement or error metric	Segmentation only (Dice/HD/IoU). No LVEF agreement metric reported despite the title.
Doan 2023	Efficacy of Artificial Intelligence Software in the Automated Analysis of Left Ventricular Function in Echocardiography in Central Vietnam. Acta Informatica Medica. 10.5455/aim.2024.32.32-36	No eligible continuous LVEF agreement or error metric	Philips QLAB auto-EF vs. manual QLAB EF in 136 pts; only correlation (r), sensitivity/specificity reported; no MAE/MAD/RMSE/bias/LoA anywhere. Reference method (Simpson vs other) also unclear.
Ge 2020	K-Net: Integrate Left Ventricle Segmentation and Direct Quantification of Paired Echo Sequence. IEEE Transactions on Medical Imaging. 10.1109/tmi.2019.2955436	No eligible continuous LVEF agreement or error metric	K-Net direct quantification of areas/dimensions/regional wall thickness on 50-subject set; no LVEF output/agreement.
He 2023	Blinded, randomized trial of sonographer versus AI cardiac function assessment. Nature. 10.1038/s41586-023-05947-3	No eligible continuous LVEF agreement or error metric	No agreement metric between stand-alone AI LVEF and an independent reference standard: the comparator (final cardiologist LVEF) was produced by reviewing and adjusting the AI's own initial annotations, and the secondary comparison assessed the AI-assisted card.
Kim 2025	EchoFM: Foundation Model for Generalizable Echocardiogram Analysis. IEEE Transactions on Medical Imaging. 10.1109/tmi.2025.3580713	No eligible continuous LVEF agreement or error metric	LVEF reported only as R2; no MAE/RMSE/bias/LoA.
Li 2024	Diagnostic efficacy of artificial intelligence model based on yolox framework integrating left ventricular segmentation and key point detection to automatically measure left ventricular ejection function in patients with chronic renal failure. Chinese Journal of Ultrasonography. 10.3760/cma.j.cn131148-20231214-00291	No eligible continuous LVEF agreement or error metric	Agreement with the manual reference reported only as correlation and ICC; no MAE, RMSE, Bland-Altman bias or limits of agreement.
Li 2025	An automatic and real-time echocardiography quality scoring system based on deep learning to improve reproducible assessment of left ventricular ejection fraction. Quantitative Imaging in Medicine and Surgery. 10.21037/qims-24-512	No eligible continuous LVEF agreement or error metric	AI-versus-expert LVEF agreement reported only as intraclass correlation, Pearson correlation and group comparisons; no MAE, RMSE, Bland-Altman bias or limits of agreement.
Szasz 2026	Automated analysis of echocardiographic images: do different artificial intelligence tools provide concordant measurements?. International Journal of Cardiovascular Imaging. 10.1007/s10554-025-03539-y	No eligible continuous LVEF agreement or error metric	Only inter-software (AI tool versus AI tool) and inter-reader (expert versus expert) agreement reported; no comparison of AI LVEF with a reference standard.
Zhang 2024	Development of automated neural network prediction for echocardiographic left ventricular ejection fraction. Frontiers in Medicine. 10.3389/fmed.2024.1354070	No eligible continuous LVEF agreement or error metric	Only a correlation coefficient (Pearson r) is reported for LVEF; no MAE, RMSE, Bland-Altman bias, or limits of agreement are reported anywhere in the text, tables, or figures.
Zhou 2023	DSANet: Dual-Branch Shape-Aware Network for Echocardiography Segmentation in Apical Views. IEEE Journal of Biomedical and Health Informatics. 10.1109/jbhi.2023.3293520	No eligible continuous LVEF agreement or error metric	Echocardiography segmentation only (DSANet); no LVEF.
Jaballah 2026	LDM-Echo: A Latent Diffusion Model for Echocardiographic Image Denoising with Clinical Evaluation. Journal of Imaging Informatics in Medicine. 10.1007/s10278-026-02168-8	LVEF quantification not the index test	Primary AI is an image-denoising diffusion model; LVEF is a downstream check using a pre-existing EF model, not an LVEF-quantification method.
O'Driscoll 2022	Left ventricular assessment with artificial intelligence increases the diagnostic accuracy of stress echocardiography. European Heart Journal Open. 10.1093/ehjopen/oeac059	LVEF quantification not the index test	AI-LVEF used as predictor for CAD detection in stress echo; no continuous LVEF agreement/performance metric vs reference standard reported.
O'Driscoll 2023	Artificial intelligence calculated global longitudinal strain and left ventricular ejection fraction predicts cardiac events and all-cause mortality in patients with chest pain. Echocardiography. 10.1111/echo.15714	LVEF quantification not the index test	AI TTE-derived LVEF is used for prognostic association only; no continuous LVEF accuracy/agreement metric versus reference standard reported.
Pettersen 2025	Real-time deep learning-based image guiding and automated left ventricular measurements to reduce test-retest variability. Open Heart. 10.1136/openhrt-2025-003783	LVEF quantification not the index test	Real-time DL guiding + automated LV measurements; outcome = test-retest variability between operator pairs; no AI-vs-human-Simpson agreement/error metric.
Riefolo 2026	Limited Contribution of Fibrosis to Myocardial Interstitial Expansion in End-Stage Cardiac Amyloidosis: Insights From Explanted Whole Hearts. Journal of the American College of Cardiology. 10.1016/j.jacc.2026.02.5128	LVEF quantification not the index test	Not an AI LVEF study (explanted amyloid heart pathology).
Sahashi 2025	Artificial Intelligence Automation of Echocardiographic Measurements. Journal of the American College of Cardiology. 10.1016/j.jacc.2025.07.053	LVEF quantification not the index test	EchoNet-Measurements: 18 linear/Doppler measurements (dimensions, wall thickness, velocities); LVEF is not a model output.
Woelfert 2026	The impact of heart rate on echocardiographic measures of left ventricular function: novel insights facilitated by deep learning. European Heart Journal. Imaging Methods and Practice. 10.1093/ehjimp/qyaf163	LVEF quantification not the index test	AI used as a measurement tool to study the effect of heart rate (atrial pacing) on LV function measures; no agreement metric between AI LVEF and a reference standard.
Yuan 2025	Understanding Transient Left Ventricular Ejection Fraction Reduction During Atrial Fibrillation With Artificial Intelligence. Journal of the American Heart Association. 10.1161/jaha.124.040641	LVEF quantification not the index test	Pretrained AI (EchoNet-Dynamic) used to re-measure LVEF in atrial fibrillation for reclassification and prognosis; no agreement metric between AI LVEF and a reference standard.
van Nierop 2026	Patient-Adaptive Echocardiography Using Cognitive Ultrasound. IEEE Transactions on Medical Imaging. 10.1109/tmi.2026.3691009	LVEF quantification not the index test	Ultrasound transmit/beamforming scheme (patient-adaptive imaging); no LVEF agreement.
Adhikari 2026	Predicting Cardiac Magnetic Resonance-Derived Ejection Fraction from Echocardiogram Via Deep Learning Approach in Tetralogy of Fallot. Pediatric Cardiology. 10.1007/s00246-025-03802-y	Population not adult	Pediatric congenital heart disease (Tetralogy of Fallot) population; not adult standard TTE study.
Li 2024	Machine Learning-Enabled Fully Automated Assessment of Left Ventricular Volume, Ejection Fraction and Strain: Experience in Pediatric and Young Adult Echocardiography. Pediatric Cardiology. 10.1007/s00246-022-03015-7	Population not adult	Pediatric/young adult echocardiography population; not a standard adult TTE study.
Lukyanenko 2026	Deep Learning-Based Automated Echocardiographic Measurements in Pediatric and Congenital Heart Disease. medRxiv. 10.64898/2026.02.06.26345782	Population not adult	Pediatric/CHD population (median age 8.5 years; external 3.5 years), not adult TTE.
Reddy 2023	Video-Based Deep Learning for Automated Assessment of Left Ventricular Ejection Fraction in Pediatric Patients. Journal of the American Society of Echocardiography. 10.1016/j.echo.2023.01.015	Population not adult	Pediatric echocardiography study; review limited to adult TTE populations.
Zuercher 2022	Retraining an Artificial Intelligence Algorithm to Calculate Left Ventricular Ejection Fraction in Pediatrics. Journal of Cardiothoracic and Vascular Anesthesia. 10.1053/j.jvca.2022.05.004	Population not adult	Pediatric population (newborns to 18 years), not an adult TTE study.
Brindise 2022	Automated Peak Prominence-Based Iterative Dijkstra's Algorithm for Segmentation of B-Mode Echocardiograms. IEEE Transactions on Biomedical Engineering. 10.1109/tbme.2021.3123612	LVEF not computed automatically by the AI method	User-initialised segmentation (three manual clicks per scan); not a fully computer-derived contour.
Nazari 2026	Development and clinical usability assessment of a web-based AI platform for automated echocardiography analysis. Computer Methods and Programs in Biomedicine. 10.1016/j.cmpb.2026.109490	LVEF not computed automatically by the AI method	Human-in-the-loop platform; AI masks reviewed and manually adjusted before the reported agreement.
Sirjani 2022	Automatic cardiac evaluations using a deep video object segmentation network. Insights into Imaging. 10.1186/s13244-022-01212-9	LVEF not computed automatically by the AI method	Semi-automated; user delineates the first-frame mask/borders (manual tracing).
Holste 2024	PanEcho: Complete AI-enabled echocardiography interpretation with multi-task deep learning. medRxiv. 10.1101/2024.11.16.24317431	Not a primary peer-reviewed journal article	medRxiv preprint of Holste 2025 (JAMA), which is included in the review.
Roberts 2026	Reproducibility of echocardiographic measurements of left ventricular systolic function: a systematic review and meta-analysis comparing artificial intelligence and clinician estimates. European Heart Journal Digital Health. 10.1093/ehjdh/ztaf145	Not a primary peer-reviewed journal article	Systematic review/meta-analysis, not a primary AI TTE continuous LVEF quantification study.
Wang 2024	EchoGPK: A Lightweight Graph Convolutional Analysis Method for Echocardiography Based on Prior Knowledge Guidance. Tien Tzu Hsueh Pao/Acta Electronica Sinica. 10.12263/dzxb.20231065	Non-English full text	Full text in Chinese (Acta Electronica Sinica); English abstract only.
Citation searching			
Bolviken 2023	Simultaneous segmentation of all four chambers in cardiac ultrasound images. Computer Methods in Biomechanics and Biomedical Engineering: Imaging & Visualization. 10.1080/21681163.2022.2073913	Not standard 2D TTE	3D echocardiography input; manual initialisation step; reference is vendor software with manual editing.
Karuzas 2025	Artificial intelligence implementation in automated heart chambers quantification during pharmacological stress echocardiography. 10.1093/ehjdh/ztaf121	Not standard 2D TTE	Stress echocardiography protocol (rest/stress phases), not standard TTE.
Komanek 2023	Quantification of left ventricular ejection fraction and cardiac output using a novel semi-automated echocardiographic method: a prospective observational study in coronary artery bypass patients. 10.1186/s12871-023-02025-z	Not standard 2D TTE	Reference standard is transoesophageal echocardiography; semi-automated non-AI AutoEF.
Li 2020	Direct estimation of left ventricular ejection fraction via a cardiac cycle feature learning architecture. 10.1016/j.compbiomed.2020.103659	Not standard 2D TTE	Distinct paper (Comput Biol Med 118:103659) with cine CMR input (non-echocardiographic input).
Tseng 2024	Real-Time Automatic M-Mode Echocardiography Measurement With Panel Attention. 10.1109/jbhi.2024.3413628	Not standard 2D TTE	RAMEM: real-time instance segmentation on M-mode echocardiograms (not standard 2D B-mode); measurement MAE refers to M-mode dimensional indices (reference M-mode measurements); no 2D Simpson LVEF agreement.
Ali 2021	Echocardiographic image segmentation using deep Res-U network. 10.1016/j.bspc.2020.102248	No eligible continuous LVEF agreement or error metric	Segmentation only (Dice 0.97); no LVEF agreement metric.
Arafati 2020	Generalizable fully automated multi-label segmentation of four-chamber view echocardiograms based on deep convolutional adversarial networks. 10.1098/rsif.2020.0267	No eligible continuous LVEF agreement or error metric	A4C segmentation; only EDV/ESV agreement (bias/LoA); no LVEF reported.
Ge 2019	PV-LVNet: Direct left ventricle multitype indices estimation from 2D echocardiograms of paired apical views with deep neural networks. 10.1016/j.media.2019.101554	No eligible continuous LVEF agreement or error metric	PV-LVNet estimates LV dimensions, areas and volume only; no LVEF reported.
Kusunose 2020	Clinically Feasible and Accurate View Classification of Echocardiographic Images Using Deep Learning. 10.3390/biom10050665	No eligible continuous LVEF agreement or error metric	Biomolecules paper: view classification CNN; EF prediction only shown as correlation under 1.9% mislabelled training data (no MAE/bias/LoA).
Maani 2024	SimLVSeg: Simplifying Left Ventricular Segmentation in 2-D+Time Echocardiograms With Self- and Weakly Supervised Learning. 10.1016/j.ultrasmedbio.2024.08.023	No eligible continuous LVEF agreement or error metric	SimLVSeg video segmentation; Dice/HD only; no LVEF agreement metric.
Qu 2024	Contour-constrained branch U-Net for accurate left ventricular segmentation in echocardiography. Medical & Biological Engineering & Computing. 10.1007/s11517-024-03201-0	No eligible continuous LVEF agreement or error metric	Segmentation accuracy (Dice) only; no LVEF value or agreement metric.
Shiokawa 2024	Accuracy and Efficacy of Artificial Intelligence-Derived Automatic Measurements of Transthoracic Echocardiography in Routine Clinical Practice. 10.3390/jcm13071861	No eligible continuous LVEF agreement or error metric	No LVEF agreement metric.
Wang 2026	Echocardiography segmentation via inter-frame fusion based diffusion network. 10.1016/j.eswa.2026.132287	No eligible continuous LVEF agreement or error metric	Generic semi-supervised contrastive regression benchmarked on non-echo datasets (IXI, AgeDB, UTK); echo EF only correlation (r 0.765), no MAE/bias vs. reference.
Zamzmi 2022	Real-time echocardiography image analysis and quantification of cardiac indices. 10.1016/j.media.2022.102438	No eligible continuous LVEF agreement or error metric	Multi-task echo pipeline (retrieval, segmentation, quantification); EF vs. expert EF shown only as correlation/Bland-Altman plot (Fig. 15) with no numeric MAE/bias/LoA in text.
Zhu 2026	Bilateral lightweight network for left ventricle segmentation in two-dimensional echocardiography. 10.1016/j.bspc.2025.109139	No eligible continuous LVEF agreement or error metric	EF agreement reported only as correlation and mean absolute percentage error (relative), no metric in EF points.
Yuan 2021	Systematic Quantification of Sources of Variation in Ejection Fraction Calculation Using Deep Learning. 10.1016/j.jcmg.2021.06.018	LVEF quantification not the index test	JACC Imaging research letter on simulated tracing/frame-selection errors in manual single-plane LVEF; no AI index test.
Abazid 2018	Visual versus fully automated assessment of left ventricular ejection fraction. 10.4103/ajm.ajm_209_17	LVEF not computed automatically by the AI method	Border correction by sonographers permitted and not separated; vendor speckle-tracking Auto-EF.
Franchi 2018	Assessment of left ventricular ejection fraction in critically ill patients at the time of speckle tracking echocardiography: intensivists in training for echocardiography versus experienced operators. 10.23736/s0375-9393.18.12249-8	LVEF not computed automatically by the AI method	Semi-automatic speckle-tracking Auto-EF requiring operator input; not an AI-based method; ICU bedside.
Hirata 2024	Reducing echocardiographic examination time through routine use of fully automated software: a comparative study of measurement and report creation time. 10.1007/s12574-023-00636-6	LVEF not computed automatically by the AI method	AI measurements manually adjusted; LVEF agreement by ICC only.
Hovnanians 2017	Validity of automated measurement of left ventricular ejection fraction and volume using the Philips EPIQ system. 10.1111/echo.13705	LVEF not computed automatically by the AI method	Borders editable at reader discretion and edited results not separated.
Sakamoto 2025	Artificial Intelligence-Based Automated Echocardiographic Analysis and the Workflow of Sonographers: A Randomized Crossover Trial (AI-Echo RCT). 10.1161/jaha.125.045637	LVEF not computed automatically by the AI method	Randomised crossover workflow trial; 'reference' = final report values after sonographer/echocardiologist verification and modification of AI output (not independent of AI), AI-assisted reference; LVEF (2D disk) concordance ICC 0.92, mean abs modification 3.9.
Al-Alusi 2026	Multi-view deep learning for echocardiogram interpretation. 10.1038/s44161-026-00780-z	Not a primary peer-reviewed journal article	News & Views commentary on another study (Barrios et al.); not a primary study; categorical outcomes.
Asch 2019	ACCURACY AND REPRODUCIBILITY OF A NOVEL ARTIFICIAL INTELLIGENCE DEEP LEARNING-BASED ALGORITHM FOR AUTOMATED CALCULATION OF EJECTION FRACTION IN ECHOCARDIOGRAPHY. 10.1016/s0735-1097(19)32053-4	Not a primary peer-reviewed journal article	Conference abstract (JACC supplement poster); also sonographers manually selected apical clips.
Balasubramani 2023	Accurate Left Ventricle Segmentation and Ejection Fraction Estimation with Deep Learning-Based Echocardiography. 10.2139/ssrn.4629020	Not a primary peer-reviewed journal article	SSRN preprint ('not peer reviewed'); also segmentation (IoU) with no LVEF agreement metric.

Supplementary Material S2. Risk of bias and applicability assessment (Tailored QUADAS-2)

This supplement gives the tailored QUADAS-2 form, decision rules and assessment process, and the judgements for all 108 included studies. The signalling questions and decision rules are reproduced from the assessment protocol, which was fixed before assessment began.

S2.1 Choice of Tool

Each included study compared an AI-derived LVEF value with a reference measurement in the same participants, the index-versus-reference design that QUADAS-2 assesses. QUADAS-2 expects its signalling questions to be tailored to each review, and it was tailored as described below for an AI index test and a continuous agreement outcome. PROBAST and its AI extension were not used because they assess prediction models rather than agreement between measurements, and QUADAS-AI had not been finalised at the time of assessment.

S2.2 Pre-specified Tailoring: Two Adaptations Were Agreed in Advance and Applied to All Studies

Index test: Because a continuous LVEF output has no diagnostic threshold, the threshold question was replaced by a question on data leakage, that is, whether the test set was independent of the data used to develop the model (SQ2.2).

Reference standard: Each reference was judged on how it was obtained and read, not on its modality, so an echocardiographic reference was not rated down solely because it was not CMR.

S2.3 Assessment Process

The form was piloted on five studies covering different reference standards, public and clinical cohorts and sample sizes, and the pilot was used to refine the decision rules. Two reviewers then independently assessed each study against its full text, and disagreements were resolved at a consensus meeting. Items not reported in a paper were rated unclear. Studies identified by the updated search and by citation searching were assessed with the same form, rules, and process.

A domain was rated low risk only if all its signalling questions were answered in the low-risk direction, high risk if any answer indicated bias, and unclear if missing information could plausibly introduce bias. Applicability was rated for domains 1 to 3. Risk of reporting bias and certainty of evidence were not formally assessed, as no meta-analysis was performed.

S2.4 Tailored Signalling Questions and Decision Anchors

**Table 6:** Tailored QUADAS-2 signalling questions and decision anchors. QUADAS-2, Quality Assessment of Diagnostic Accuracy Studies 2; LVEF, left ventricular ejection fraction; TTE, transthoracic echocardiography; EF, ejection fraction; CMR, cardiac magnetic resonance.

Signalling question	Decision anchor (Low/High/Unclear)
Domain 1. Patient selection	
SQ1.1 Consecutive or random sample	Low: stated consecutive/random, or full public dataset used in its entirety. High: selective/convenience sampling. Unclear: not described.
SQ1.2 Case-control/enriched design avoided	High: cohort artificially balanced across EF strata or otherwise enriched so as to distort the clinical spectrum. Low: natural spectrum or unselected dataset.
SQ1.3 Inappropriate exclusions avoided	High: poor-quality/unanalysable images or specific EF ranges excluded so as to inflate agreement. Low: minimal or justified exclusions.
Applicability	High concern if a narrow single-disease or single-setting subgroup, or a population not generalisable to adults undergoing standard TTE for any indication.
Domain 2. Index test (AI model)	
SQ2.1 Interpreted without knowledge of the reference	Low: automated output with no tuning to the reference. High: model tuned on the test set.
SQ2.2 Pre-specified model; test set independent of training (no leakage)	Low: training and test data separated at patient level, with all examinations of a patient in the same partition (including predefined public-dataset splits), or an external test set. High: patient overlap between training and test data, a headline result from k-fold cross-validation without a separate test set, or test data used for tuning or model selection. Unclear: separation not described, or possible overlap of the test cohort with development data. Overfitting was judged through these items.
Applicability	High concern if the index test requires non-standard preprocessing or manual steps beyond the standard clinical workflow (selection of the view, loop or end-diastolic and end-systolic frames is part of that workflow).
Domain 3. Reference standard	
SQ3.1 Reference likely to classify LVEF correctly (per-study judgement)	Low: rigorous reference (CMR; core laboratory; Simpson’s biplane by a trained reader; averaged multi-reader). High: single unblinded reader, visual estimate alone, or undefined method. Unclear: method not stated, including routine clinical or dataset labels whose method and readers are not reported.
SQ3.2 Reference interpreted without knowledge of the AI output	Low: independent or pre-existing labels. High: read with the AI output visible. Unclear: not stated.
Applicability	High concern if the reference is itself a semi-automated or AI method of unvalidated accuracy.
Domain 4. Flow and timing	
SQ4.1 Appropriate interval between index and reference	Low: both derived from the same study or images. High: temporally separated acquisitions risking physiological change.
SQ4.2 All patients received a reference standard	High if the reference was applied to only a subset.
SQ4.3 All patients received the same reference standard	High if mixed references were applied non-systematically. Unclear if test sets carried reference labels of different origin.
SQ4.4 All enrolled patients included in the analysis	Low: all accounted for, or attrition explained. High: unexplained dropouts, or cases the AI could not analyse removed from the agreement analysis.

S2.5 Study-Level Judgements

**Table 7:** Risk of bias and applicability judgements for the 108 included studies. L, low; H, high; U, unclear. D1, patient selection; D2, index test; D3, reference standard; D4, flow and timing; suffix a, applicability (rated for D1 to D3 only). A reason is given for each rating other than low; the cell is blank where all ratings were low. LVEF, left ventricular ejection fraction; CMR, cardiac magnetic resonance; EF, ejection fraction; HFpEF, heart failure with preserved ejection fraction; STEMI, ST-elevation myocardial infarction.

Study	D1	D1a	D2	D2a	D3	D3a	D4	Reason for ratings other than low
Akan, 2025 [24]	L	L	L	L	U	L	U	D3 unclear: EchoNet-Dynamic routine clinical labels. D4 unclear: test set size reported inconsistently.
Akbari, 2024 [25]	L	L	H	L	L	L	L	D2 high: 10-fold cross-validation, no separate test set.
Ali, 2025 [26]	L	L	L	L	U	L	L	D3 unclear: EchoNet-Dynamic routine clinical labels.
Alven, 2024 [27]	H	H	L	L	H	L	H	D1 and D1a high: cohort balanced to 50% reduced EF. D3 high: reference mostly visual estimation. D4 high: reference methods applied non-systematically.
Anisuzzaman, 2025 [28]	L	L	U	L	U	L	U	D2 unclear: internal test cohort from the development institution; patient-level separation not explicit. D3 and D4 unclear: hierarchical clinical reference; readers not reported.
Anisuzzaman, 2026 [29]	L	L	U	L	U	L	U	D2 unclear: patient-level separation not stated. D3 and D4 unclear: hierarchical clinical reference, readers not reported.
Antonaci, 2026 [30]	H	H	H	L	U	L	L	D1 high: convenience sample of 22 healthy volunteers and 5 selected patients. D1a high: mostly healthy volunteers. D2 high: most test patients were also used for fine-tuning. D3 unclear: clinician-reported EF, method not stated.
Anwaar, 2025 [31]	H	L	L	L	L	L	L	D1 high: poor-quality test images excluded.
Artman, 2025 [32]	L	L	L	L	U	L	U	D3 unclear: EchoNet-Dynamic routine clinical labels. D4 unclear: test sets with labels of different origin.
Asch, 2019 [35]	U	L	L	L	L	L	L	D1 unclear: sampling not described.
Asch, 2021 [34]	U	L	L	L	L	L	L	D1 unclear: sampling not described.
Asch, 2022 [33]	H	H	L	L	L	L	U	D1 high: only acceptable-quality views included. D1a high: hospitalised COVID-19 patients only. D4 unclear: 476 of 870 patients had both measurements.
Batool, 2023 [37]	L	L	L	L	U	L	L	D3 unclear: EchoNet-Dynamic routine clinical labels.
Batool, 2024 [38]	L	L	L	L	U	L	L	D3 unclear: EchoNet-Dynamic routine clinical labels.
Batool, 2025 [36]	L	L	L	L	U	L	U	D3 unclear: EchoNet-Dynamic routine clinical labels. D4 unclear: two datasets with labels of different origin.
Belfilali, 2022 [39]	L	L	H	L	L	L	L	D2 high: k-fold cross-validation, no separate test set.
Blaivas, 2022 [40]	L	L	L	L	L	L	U	D4 unclear: some videos excluded without full accounting.
Blixt, 2026 [54]	H	L	L	L	L	L	L	D1 high: cases selected into predefined groups; arrhythmia and valve disease excluded.
Cai, 2024 [41]	U	L	L	L	L	L	L	D1 unclear: sampling not described.
Chen, 2022 [42]	L	H	L	L	L	L	L	D1a high: dilated left ventricle and heart failure cohort only.
Cheng, 2025 [43]	L	L	L	L	U	L	U	D3 unclear: EchoNet-Dynamic routine clinical labels. D4 unclear: external test labels of different origin.
Chiruchuri, 2026 [44]	L	L	L	L	U	L	L	D3 unclear: EchoNet-Dynamic routine clinical labels.
Christensen, 2024 [45]	U	L	L	L	U	L	L	D1 unclear: sampling not described. D3 unclear: reference from routine clinical reports.
Cotella, 2023 [46]	H	H	L	L	L	L	L	D1 high: 8 of 59 examinations excluded for image quality. D1a high: amyloidosis cohort only.
Cui, 2022 [47]	L	L	H	L	L	L	L	D2 high: 10-fold cross-validation, no separate test set.
Dai, 2023 [48]	L	L	L	L	U	L	U	D3 unclear: EchoNet-Dynamic routine clinical labels. D4 unclear: test set limited to videos with available labels.
Dong, 2026 [49]	L	L	L	L	L	L	U	D4 unclear: datasets with labels of different origins combined, and some unusable videos excluded.
Dowsing, 2026 [50]	L	H	L	L	L	L	L	D1a high: cardio-oncology surveillance only.
Er, 2025 [51]	U	L	L	L	U	L	U	D1 and D4 unclear: test set filtered to 1,209 videos; criteria not fully reported. D3 unclear: EchoNet-Dynamic routine clinical labels.
Fatima, 2024 [52]	L	L	L	L	L	L	L	
Fisher, 2026 [53]	L	L	U	L	U	L	L	D2 unclear: patient-level separation not stated. D3 unclear: cardiologist-reported EF, method not stated.
Ghorbani, 2020 [55]	L	L	L	L	U	L	L	D3 unclear: clinical-reported EF, method not reported.
Gladding, 2021 [56]	H	H	L	L	U	L	H	D1 high: case-control design (heart failure with reduced EF versus controls). D1a high: selected groups. D3 unclear: single sonographer, blinding not stated. D4 high: 18% of AI values excluded as non-physiological.
Gul, 2026 [57]	H	H	U	H	H	H	H	D1 high: image exclusions. D1a high: single-centre ischaemic heart disease cohort. D2 unclear and D4 high: test set size reported inconsistently (13 versus 165 patients). D2a high: videos manually trimmed and flipped before analysis in a custom interface. D3 high: single-reader reference. D3a high: reference based on segmentations by observers after a two-month training course, confirmed by one cardiologist.
Hauptmann, 2025 [58]	L	L	H	L	U	L	U	D2 high: six-fold cross-validation, no separate test set. D3 unclear: single-plane apical four-chamber reference. D4 unclear: echocardiograms available for 2,466 of 3,289 participants, and the number analysed for LVEF not fully accounted for.
Holste, 2025 [59]	L	L	L	L	U	L	U	D3 unclear: clinical-report reference. D4 unclear: several exclusions in the flow of participants.
Hu, 2025 [60]	U	L	L	L	U	L	U	D1 unclear: sampling not described. D3 unclear: reference by 1 of 18 operators of varying experience. D4 unclear: some studies excluded for technical issues.
Iwasaki, 2025 [61]	U	L	L	L	L	L	L	D1 unclear: random subsampling, sampling frame not fully described.
Jang, 2024 [62]	L	H	L	L	L	L	L	D1a high: revascularised STEMI cohort only.
Jiang, 2023 [43]	L	H	L	L	U	L	U	D1a high: cardio-oncology surveillance cohort. D3 unclear: reader numbers not reported. D4 unclear: technical success below 100%.
Kim, 2022 [64]	H	H	U	L	L	L	L	D1 high: heart failure, coronary and valve disease, and uninterpretable images excluded. D1a high: young, structurally normal population. D2 unclear: separation of test data unclear.
Knackstedt, 2015 [65]	U	L	L	L	L	L	L	D1 unclear: initial sampling frame not fully described.
Lafitte, 2025 [66]	L	L	L	L	U	L	L	D3 unclear: reference by operators of mixed experience.
Lau, 2023 [67]	L	L	L	L	U	L	U	D3 unclear: clinical and public-dataset labels. D4 unclear: exclusions across datasets.
Laumer, 2022 [68]	L	L	H	L	U	L	L	D2 high: 10-fold cross-validation, no separate test set. D3 unclear: EchoNet-Dynamic routine clinical labels.
Leclerc, 2019 [11]	L	L	H	L	L	L	L	D2 high: 10-fold cross-validation, no separate test set.
Leclerc, 2020 [69]	L	L	H	L	L	L	L	D2 high: 10-fold cross-validation, no separate test set.
Li, 2020 [72]	U	L	L	L	L	L	L	D1 unclear: sampling and exclusions not described.
Li, 2020 [73]	L	L	L	L	L	L	L	
Li, 2023 [70]	L	L	H	L	L	L	L	D2 high: 5-fold cross-validation, no separate test set.
Li, 2025 [71]	U	L	L	L	U	L	U	D1 and D4 unclear: test set of 1,003 of 1,277 videos, reason not stated. D3 unclear: EchoNet-Dynamic routine clinical labels.
Li, 2025 [74]	L	L	L	L	U	L	L	D3 unclear: public-dataset labels.
Lin, 2022 [77]	H	L	L	L	U	L	U	D1 high: about 40% of studies excluded for image quality. D3 unclear: clinical-report reference. D4 unclear: large attrition.
Lin, 2024 [75]	L	L	H	L	U	L	U	D2 high: 5-fold cross-validation, no separate test set. D3 unclear: EchoNet-Dynamic routine clinical labels. D4 unclear: two datasets with labels of different origin.
Lin, 2025 [76]	L	L	L	L	U	L	U	D3 unclear: manual LVEF method not named. D4 unclear: AI input and reference from different scanners; interval not stated.
Liu, 2020 [79]	L	L	L	L	U	L	U	D3 unclear: EchoNet-Dynamic routine clinical labels. D4 unclear: two datasets with labels of different origin.
Liu, 2021 [78]	U	L	L	L	L	L	L	D1 unclear: sampling not described.
Malins, 2025 [80]	L	L	U	L	U	L	U	D2 unclear: patient-level separation not fully explicit. D3 and D4 unclear: hierarchical clinical reference.
McMenamin, 2026 [81]	L	H	L	L	L	L	U	D1a high: older Black and Caribbean Hispanic patients with heart failure screened for cardiac amyloidosis. D4 unclear: AI measurement yield 79% to 93%, unmeasurable values treated as missing.
Meng, 2024 [82]	L	L	L	L	U	L	U	D3 unclear: EchoNet-Dynamic routine clinical labels. D4 unclear: two datasets with labels of different origin.
Merin, 2026 [83]	L	L	L	L	H	L	L	D3 high: reference was visual expert estimation.
Mo, 2025 [84]	L	L	L	L	U	L	U	D3 unclear: EchoNet-Dynamic routine clinical labels. D4 unclear: attrition not fully accounted for.
Moal, 2022 [85]	U	L	L	L	L	L	U	D1 and D4 unclear: exclusions and attrition not fully reported.
Molek-Dziadosz, 2025 [86]	L	L	L	L	L	L	H	D4 high: CMR and echocardiography up to 7 days apart.
Mor-Avi, 2023 [87]	H	H	L	L	L	L	L	D1 high: major abnormalities excluded, sinus rhythm only. D1a high: 12 normal hearts.
Myhre, 2024 [88]	L	L	L	L	L	L	L	
Nagata, 2025 [89]	L	L	L	L	L	L	L	
Olaisen, 2024 [90]	L	L	L	L	U	L	H	D3 unclear: reader numbers vary across datasets. D4 high: reference handled differently across the four datasets.
Ono, 2022 [91]	U	U	L	L	L	L	L	D1 and D1a unclear: small cohort (29), mostly healthy volunteers.
Ouyang, 2020 [10]	L	L	L	L	U	L	L	D3 unclear: clinical label method not reported.
Painchaud, 2022 [92]	U	L	U	L	L	L	L	D1 unclear: selection of the 98-sequence subset not described. D2 unclear: data split not fully described.
Pellikka, 2022 [93]	L	L	L	L	H	L	H	D3 high: clinical LVEF by mixed methods including visual estimation. D4 high: methods applied non-systematically.
Sabo, 2025 [94]	L	L	L	L	L	L	L	
Samtani, 2022 [95]	L	L	L	L	L	L	L	
Schneider, 2021 [96]	L	U	L	L	L	L	L	D1a unclear: 14-patient pilot with images acquired by novices.
Sfakianakis, 2023 [97]	L	L	L	L	L	L	L	
Shen, 2025 [98]	U	H	H	H	L	L	U	D1 unclear: how the 127 patients were selected is not described. D1a and D2a high: non-standard rotational acquisition with 3D reconstruction. D2 high: no conventional held-out test set. D4 unclear: patient flow and exclusions not reported.
Singh, 2024 [99]	L	L	L	L	U	L	U	D3 unclear: EchoNet-Dynamic routine clinical labels. D4 unclear: minor frame and sample exclusions.
Smistad, 2020 [100]	U	L	L	L	L	L	L	D1 unclear: sampling of the 100-patient subset not fully specified.
Sun, 2026 [101]	H	L	L	L	L	L	L	D1 high: purposive sampling enriched for aortic regurgitation, LVEF range, atrial fibrillation, and poor image quality.
Sveric, 2023 [102]	L	L	L	L	U	L	L	D3 unclear: reader numbers not reported.
Sveric, 2024 [103]	L	L	L	L	L	L	L	
Szijarto, 2025 [104]	U	L	L	L	L	L	L	D1 unclear: sampling not described.
Tromp, 2022 [105]	L	L	L	L	U	L	L	D3 unclear: reference method and readers vary across cohorts.
Tromp, 2022 [106]	U	U	L	L	L	L	L	D1 unclear: sampling not described. D1a unclear: heart failure-enriched trial cohort.
Van, 2025 [107]	L	L	L	L	L	L	L	
Vega, 2024 [108]	U	L	L	L	U	L	H	D1 and D3 unclear: sampling and reference method not standardised across clinics. D4 high: reference methods differ across clinics.
Venema, 2026 [109]	H	H	U	L	L	L	H	D1 high: enriched design with heart failure, at-risk and control groups, and one cohort limited to patients measurable by both methods. D1a high: HFpEF-focused populations. D2 unclear: possible overlap of test cohorts with the commercial tool's development data. D4 high: cases without an AI value removed from the analysis.
Vukadinovic, 2025 [110]	L	L	L	L	U	L	L	D3 unclear: clinician-reported LVEF, method not specified.
Vyver, 2024 [111]	L	L	L	L	L	L	L	
Wan, 2024 [112]	L	L	H	L	U	L	U	D2 high: 5-fold cross-validation, no separate test set. D3 unclear: EchoNet-Dynamic routine clinical labels. D4 unclear: two datasets with labels of different origin.
Wang, 2022 [113]	L	H	L	L	U	L	L	D1a high: COVID-19 inpatients only. D3 unclear: reader numbers not reported.
Wei, 2022 [114]	H	L	L	L	L	L	L	D1 high: 10 poor-quality test cases excluded.
Wu, 2025 [117]	L	L	U	L	L	L	L	D2 unclear: train and test split not described.
Wu, 2025 [116]	L	L	L	L	U	L	L	D3 unclear: EchoNet-Dynamic routine clinical labels.
Wu, 2026 [115]	L	L	L	L	L	L	U	D4 unclear: labels of different origin across datasets.
Xue, 2022 [118]	L	L	H	L	L	L	L	D2 high: 10-fold cross-validation, no separate test set.
Yamaguchi, 2023 [119]	L	L	L	L	L	L	L	
Yang, 2024 [120]	L	L	L	L	U	L	L	D3 unclear: EchoNet-Dynamic routine clinical labels.
Zeng, 2023 [121]	L	L	L	L	U	L	U	D3 unclear: EchoNet-Dynamic routine clinical labels. D4 unclear: test set of 1,226 of 1,277 videos.
Zhang, 2018 [122]	U	L	L	L	U	H	U	D1, D3 and D4 unclear: sampling, reference method and attrition not fully reported. D3a high: reference partly from semi-automated software of unvalidated accuracy.
Zhang, 2022 [123]	L	L	L	L	U	L	U	D3 unclear: physician-reported single-plane EF, recorded as discrete values. D4 unclear: variable proportion removed by quality control.
Zhang, 2026 [124]	L	L	L	L	U	L	U	D3 unclear: public-dataset labels. D4 unclear: several datasets.
Zhang, 2026 [125]	L	L	L	L	L	L	L	
Zhou, 2026 [127]	L	L	L	L	U	L	U	D3 unclear: EchoNet-Dynamic routine clinical labels. D4 unclear: two datasets with labels of different origin.
Zhou, 2026 [126]	L	L	U	L	U	L	L	D2 unclear: custom split of frame pairs, patient separation not stated. D3 unclear: EchoNet-Dynamic routine clinical labels.
Zhu, 2024 [128]	L	L	L	L	L	L	L	
Zhu, 2025 [129]	H	H	L	L	L	L	L	D1 high: 67% excluded for image quality, incomplete data, or hypertension. D1a high: cohort selected to balance age across left ventricular geometry groups.

S2.6 Distribution of Judgements

**Table 8:** Number of studies by judgement (n = 108). Low risk in all four domains: 17. High risk in ≥1 domain: 32. High concern in ≥1 applicability item: 18 studies.

Domain	Low	High	Unclear
D1 risk of bias	73	15	20
D1 applicability concern	88	17	3
D2 risk of bias	85	13	10
D2 applicability concern	106	2	0
D3 risk of bias	54	4	50
D3 applicability concern	106	2	0
D4 risk of bias	65	8	35

Supplementary Material S3. Extraction dataset

The extraction dataset is provided as the accompanying Excel file Supplementary_Material_S3_dataset.xlsx. The Study_level sheet gives one headline result for each of the 108 studies, with study characteristics, the provenance of each value, approximate 95% confidence intervals for the limits of agreement, and the QUADAS-2 judgements. The Cohort_level sheet gives every eligible model, cohort and reference comparison (181 results). The Summary_formulas sheet reproduces the main summaries and counts reported in the manuscript with formulas, and the External_by_study, External_priority, and All_results_by_study sheets reproduce the analyses of external results and of all results. Subgroup and sensitivity summaries are reproduced by the analysis code (Supplementary Material S4). All metrics are in EF percentage points, and each sheet and variable is defined in the README sheet. The dataset and code are available at: https://doi.org/10.5281/zenodo.23290708.

Supplementary Material S4. Analysis code

The analysis code (Python) reproduces the study-level summaries (Table 2), the sensitivity and external analyses, the Spearman correlations with test cohort size, and Figures 2, 3 from Supplementary Material S3. The dataset and code are available at: https://doi.org/10.5281/zenodo.23290708. It requires Python 3 with pandas, numpy, scipy, openpyxl, and matplotlib, and is run as: python Supplementary_Material_S4_analysis_code.py Supplementary_Material_S3_dataset.xlsx [output folder]. All summaries are unweighted study-level medians with interquartile ranges, and nothing is pooled.

Supplementary Material S5. Study-level mean absolute error
